# Supplementary material for: Genome-wide identification and expression analysis of NADPH oxidase genes in response to ABA and abiotic stresses, and in fibre formation in Gossypium
Source: PeerJ. 2020 Jan 17;8:e8404. doi: 10.7717/peerj.8404 (PMC6970565; doi:10.7717/peerj.8404)
Supplement: Supplemental Information 1 [file peerj-08-8404-s001.doc]

Table S1 The gene primers used for quantitative real-time RT-PCR experiments

| Genes | Gene number | Forward primers (5ʹ-3ʹ) | Reverse primers (5ʹ-3ʹ) |
| --- | --- | --- | --- |
| *GhUBQ7* | Gh_A11G0969 | GAAGGCATTCCACCTGACCAAC | CTTGACCTTCTTCTTCTTGTGCTTG |
| *GhRbohBA* | Gh_A11G2426 | GGTTGGACTGGCGTGAC | GGGCATAGACAATAATAAATA |
| *GhRbohBD* | Gh_D11G2743 | AGGGGGGTAATGAATGAGGT | TCCGACTCTCTTGTCTGTATGG |
| *GhRbohD-1A* | Gh_A05G1666 | AAAGGAAGTGCGGAGACTCTTA | CAACAGCAATAACCTTGTGGAA |
| *GhRbohD-1D* | Gh_D05G1864 | GGCTTGGAGAACGGGAACTA | TGGCGTCTCCTTCCTCATAC |
| *GhRbohD-2A* | Gh_A01G0943 | CACTTTGCGAAGCCTAATTG | CCGAAGCTAGATGACGTAGTTCT |
| *GhRbohD-2D* | Gh_D01G0990 | AAAATGGCGTCGATGTCGT | AATCCAAAGCTAGATGGCGTA |
| *GhRbohD-3A* | Gh_A05G2211 | AGGGCAGAAGGAAACAATACTA | TCCCTTGTCACCCAGTAAAAG |
| *GhRbohD-3D* | Gh_D05G2471 | TCAGGGCAGAAGGAGACAACAC | TTGCTCCCTTGTCACCCAGTAA |
| *GhRbohEA* | Gh_A07G0143 | GAAGGGGATGGATCTTGTTG | TAGTGGAACGAAGCCAAGTC |
| *GhRbohED* | Gh_D07G0136 | GGCGAAGGGGATGGGTTTT | TTGAGCTTGAGTGTCTCGGCAGA |
| *GhRbohF-1A* | Gh_A02G1791 | AGCGTGTACGAGGAAGGAGA | TAAAACACTCCTGCTATTCTTGC |
| *GhRbohF-1D* | Gh_D03G0688 | CAAATGCTTACTTTTATTGGGT | CAGTAAAACACTCCTATTCTTGC |
| *GhRbohF-2A* | Gh_A08G0982 | TTTGAATGCGTGTACGAGGA | CTATGGAAATGATTTAATAGCGAT |
| *GhRbohF-2D* | Gh_D08G1257 | CAATGATTCAGGCACTCCCAAC | GCAAAATGAGTCCGCACCCT |
| *GhRbohF-3A* | Gh_A12G2669 | GCCAAGAATGGAGTTGACATC | CACAATAAAAAACCCCTGCTA |
| *GhRbohF-3D* | Gh_D12G2750 | CAAGAATGGAGTTGACATCGTT | CCGCAATAAAAGACCCCTA |
| *GhRbohHA* | Gh_A07G0856 | AAAGGGCATATTTCTACTGGG | TGTTGTTATCCTGCTTTCTGAG |
| *GhRbohHD* | Gh_D07G0928 | GGCGTATTTCTATTGGGTGAC | GTGTTGTTATCCTGCTTTCTGAG |
| *GhRbohK-1A* | Gh_A07G0398 | CCAAGGAAGAACAAGAAGATG | GGAGTTTGAGAGACTGGTTTTAT |
| *GhRbohK-1D* | Gh_D07G0463 | CAACCATGCTAAGAACGGC | TATCCCACAGTAGAACACCCC |
| *GhRbohK-2A* | Gh_A12G1774 | CGCTAGCAGCACCGTGT | CAATAAGGAGAGTGTAAACA |
| *GhRbohK-2D* | Gh_D12G1932 | CACTAGCGGCACCGTGTT | AGATGCTTTGGGAGGTTGAT |
| *GhRbohK-3A* | Gh_A03G0476 | TGGGGCTACCATAAATAACAAA | GCATCACCTTCTTCGTAAACAC |
| *GhRbohK-3D* | Gh_D03G1062 | TGCCATAAATTATAAAACGAGTTC | ATCAAAAGAGCCTTGTTCCCT |
| *GhRbohLA* | Gh_A12G2653 | AAGAGGGTGATGCTCGGTCG | GTGCGTACCGGGGTTCGA |
| *GhRbohLD* | Gh_D12G1807 | AAAAGGAGGCTGTTGTAGAGG | ATTGTGTGTGTAACGGGGTTC |

Table S2 Expression profiles of 26 *GhRbohs* in distinct tissues of cotton

| Genes | Roots | Stems | Leaves | Flowers | Fibers |
| --- | --- | --- | --- | --- | --- |
| *GhRbohBA* | 1b | 0.03±0.02c | 0.02±0.01c | 1.15±0.04a | 0.01±0.01d |
| *GhRbohD-1A* | 1d | 0.12±0.11e | 4.08±0.95b | 56.49±5.69a | 2.31±0.13c |
| *GhRbohD-1D* | 1b | 0.10±0.04c | 0.04±0.01c | 3.97±0.76a | 0.08±0.01c |
| *GhRbohD-2A* | 1b | 0.03±0.01d | 0.20±0.06c | 1.62±0.02a | 0.01±0.01e |
| *GhRbohD-2D* | 1b | 0.09±0.03c | 0.09±0.01c | 1.53±0.13a | 0.22±0.11c |
| *GhRbohD-3A* | 1d | 1.27±0.14c | 3.37±0.17b | 13.20±2.09a | 0.38±0.18e |
| *GhRbohD-3D* | 1b | 0.89±0.45b | 0.06±0.02c | 12.99±2.02a | 0.07±0.03c |
| *GhRbohEA* | 1b | 0.27±0.17c | 0.07±0.01d | 22.90±4.49a | 0.29±0.11c |
| *GhRbohED* | 1b | 0.11±0.01c | 0.15±0.13c | 10.34±3.57a | 0.13±0.01c |
| *GhRbohF-1A* | 1c | 1.59±0.30b | 1.04±0.29c | 19.80±4.05a | 1.55±0.06b |
| *GhRbohF-1D* | 1c | 1.10±0.03c | 2.24±0.01b | 75.60±4.04a | 1.83±0.32b |
| *GhRbohF-2A* | 1b | 0.15±0.03d | 0.01±1.2E-4e | 7.13±0.86a | 0.75±0.15c |
| *GhRbohF-2D* | 1c | 1.56±0.32b | 1.08±0.23bc | 2.78±0.37a | 0.26±0.01d |
| *GhRbohF-3A* | 1b | 0.80±0.40b | 1.29±0.50b | 5.11±0.35a | 0.90±0.01b |
| *GhRbohF-3D* | 1c | 1.40±0.10b | 0.30±0.09d | 15.89±1.15a | 0.03±0.01e |
| *GhRbohHA* | 1c | 0.21±0.09d | 18.60±3.55b | 476.07±40.56a | 0.21±0.01d |
| *GhRbohHD* | 1c | 6.37±1.55b | 0.77±0.46c | 3444.01±1121.5a | 0.13±0.11d |
| *GhRbohK-1A* | 1b | 0.22±0.11c | 0.01±0.01d | 3.34±0.32a | 0.08±0.03c |
| *GhRbohK-2A* | 1c | 2.35±0.67b | 1.82±0.45b | 24.08±2.28a | 3.57±1.37b |
| *GhRbohK-2D* | 1c | 1.85±0.12b | 1.77±0.37b | 30.52±4.66a | 1.64±0.11b |
| *GhRbohK-3A* | 1c | 2.19±0.55b | 3.77±0.28b | 12.21±1.35a | 3.23±0.33b |
| *GhRbohK-3D* | 1c | 5.20±2.28b | 13.19±3.49a | 19.07±1.83a | 5.79±1.28b |
| *GhRbohLA* | 1b | 0.26±0.15c | 0.14±0.02c | 3.64±0.68a | 0.59±0.26c |
| *GhRbohLD* | 1b | 0.15±0.05d | 0.08±0.06d | 2.43±0.26a | 0.44±0.01c |
| *GhRbohBD* | 1a | 0.08±0.01b | 0.01±0.01c | 0.13±0.03b | 0.001±4.20E-4d |
| *GhRbohK-1D* | 1a | 0.31±0.08b | 0.049±0.03c | 0.45±0.15b | 0.13±0.08c |

Note: The relative expression of the genes in roots was set as 1. Data are presented as mean ± SE. Different lowercase letters above the error bar indicate significant differences in gene expression levels among diverse tissues by one-way ANOVA and Tukey’s HSD test (n≥3, *P* ≤ 0.05).

Table S3 Effects of ABA on the expression of *GhRbohs* in roots

| Genes | 0 h | 3 h | 6 h | 12 h | 24 h |
| --- | --- | --- | --- | --- | --- |
| *GhRbohD-1A* | 1a | 0.22±0.10c | 0.06±0.04e | 0.12±0.02d | 0.35±0.14b |
| *GhRbohD-1D* | 1a | 0.61±0.08b | 0.50±0.04c | 0.20±0.01d | 1.10±0.20a |
| *GhRbohEA* | 1b | 0.73±0.03c | 0.67±0.67cd | 0.59±0.07d | 2.31±0.54a |
| *GhRbohF-1D* | 1a | 0.42±0.06c | 0.31±0.08c | 0.67±0.04b | 0.63±0.08b |
| *GhRbohF-3A* | 1a | 0.521±0.13c | 0.34±0.03d | 0.36±0.06d | 0.836±0.01b |
| *GhRbohK-3D* | 1a | 0.14±0.04c | 0.17±0.01c | 0.91±0.09a | 0.56±0.13b |
| *GhRbohBA* | 1c | 0.73±0.17c | 2.10±0.47a | 1.30±0.07b | 0.23±0.12d |
| *GhRbohBD* | 1c | 0.91±0.13c | 1.63±0.24a | 1.2±0.04b | 0.85±0.16c |
| *GhRbohD-2A* | 1c | 6.44±1.62a | 7.43±1.88a | 0.87±0.11c | 3.34±0.34b |
| *GhRbohD-2D* | 1a | 1.03±0.09a | 0.12±0.01c | 0.69±0.07b | 0.14±0.03c |
| *GhRbohED* | 1b | 0.90±0.24b | 1.62±0.18a | 0.90±0.14b | 0.93±0.12b |
| *GhRbohF-2A* | 1d | 1.39±0.11c | 7.64±1.48a | 3.62±1.12b | 0.43±0.02e |
| *GhRbohHA* | 1a | 0.71±0.14b | 1.14±0.13a | 0.09±1E-3c | 0.66±0.13b |
| *GhRbohK-1A* | 1b | 1.58±0.19a | 0.60±0.21bc | 0.96±0.21b | 0.40±0.03c |
| *GhRbohK-1D* | 1b | 1.13±0.08a | 0.76±0.03c | 0.38±0.04d | 0.38±0.12d |
| *GhRbohLD* | 1b | 1.28±0.12a | 0.77±0.03d | 0.87±0.05c | 0.76±0.01d |
| *GhRbohD-3A* | 1a | 0.18±0.01b | 0.13±0.07b | 0.12±0.01b | 0.24±0.13b |
| *GhRbohD-3D* | 1a | 0.56±0.12b | 0.16±0.02c | 0.12±9.372E-4c | 0.20±0.08c |
| *GhRbohF-2D* | 1a | 0.47±0.47b | 0.33±0.10c | 0.16±0.07d | 0.33±0.06c |
| *GhRbohF-3D* | 1a | 0.61±0.03b | 0.31±0.03c | 0.22±0.07c | 0.31±0.11c |
| *GhRbohHD* | 1a | 0.48±0.033b | 0.49±0.05b | 0.17±0.01d | 0.30±0.06c |
| *GhRbohK-2D* | 1a | 0.69±0.20b | 0.71±0.11b | 0.67±0.06b | 0.40±0.07c |
| *GhRbohK-3A* | 1a | 0.34±0.10c | 0.80±0.12b | 0.03±0.01e | 0.13±0.01d |
| *GhRbohF-1A* | 1e | 1.79±0.07d | 3.72±0.11c | 4.81±0.78b | 8.10±1.43a |
| *GhRbohK-2A* | 1e | 2.02±0.20c | 1.43±0.18d | 5.24±1.13a | 2.98±0.24b |
| *GhRbohLA* | 1c | 1.12±0.16c | 1.33±0.13b | 1.36±0.04b | 2.13±0.39a |

Note: The relative expression of *GhRbohs* in roots was assayed after treatments with 100 µM ABA for the indicated periods of time. *GhUBQ7* was act as the internal control. The expression value at 0 h was set as 1. Data are presented as mean ± SE. Different lowercase letters above the error bar show significant differences in gene expression levels at different time points by one-way ANOVA and Tukey’s HSD test (n≥3, *P* ≤ 0.05).

Table S4 Effects of 200 mM NaCl on the expression of *GhRbohs* in roots

Note: *GhUBQ7* was used as the internal control. The expression value at 0 h was set as 1. Results are presented as mean ± SE. Different lowercase letters above the error bar represent significant differences in transcript abundances at different time points by one-way ANOVA and Tukey’s HSD test (n≥3, *P* ≤ 0.05).

| Genes | 0 h | 3 h | 6 h | 12 h | 24 h |
| --- | --- | --- | --- | --- | --- |
| *GhRbohD-2D* | 1c | 0.35±0.11d | 0.16±0.05e | 2.30±0.23a | 1.55±0.31b |
| *GhRbohF-2D* | 1a | 0.25±0.07c | 0.35±0.09c | 0.32±0.02c | 0.69±0.06b |
| *GhRbohLA* | 1a | 1.13±0.15a | 0.31±0.04b | 1.06±0.22a | 1.18±0.05a |
| *GhRbohLD* | 1a | 0.71±0.02b | 0.40±0.02c | 1.04±0.05a | 1.33±0.26a |
| *GhRbohBA* | 1b | 4.07±1.41a | 0.90±0.13b | 0.04±0.01d | 0.27±0.10c |
| *GhRbohBD* | 1b | 2.66±0.23a | 1.09±0.20b | 1.05±0.20b | 0.41±0.05c |
| *GhRbohD-1A* | 1c | 2.00±0.14a | 1.68±0.02b | 2.32±0.33a | 0.45±0.10d |
| *GhRbohD-1D* | 1c | 1.40±0.04b | 4.19±0.09a | 1.37±0.29b | 4.25±0.36a |
| *GhRbohD-2A* | 1a | 0.91±0.06a | 1.20±0.20a | 1.16±0.19a | 0.65±0.11b |
| *GhRbohD-3A* | 1 | 1.07±0.01 | 1.28±0.22 | 0.96±0.02 | 1.25±0.25 |
| *GhRbohD-3D* | 1b | 1.40±0.12a | 1.11±0.29ab | 0.91±0.10b | 0.27±0.13c |
| *GhRbohF-1D* | 1c | 1.97±0.01b | 3.92±0.15a | 0.88±0.07c | 0.75±0.04d |
| *GhRbohF-3D* | 1c | 1.42±0.15b | 1.85±0.27a | 1.15±0.14bc | 1.44±0.15b |
| *GhRbohK-1A* | 1a | 1.03±0.06a | 1.11±0.16a | 0.86±0.03b | 0.24±0.08c |
| *GhRbohK-2D* | 1c | 2.41±0.22b | 5.11±0.72a | 3.82±0.80a | 1.01±0.25c |
| *GhRbohK-3D* | 1c | 1.18±0.20c | 2.65±0.28b | 6.09±0.46a | 2.78±0.16b |
| *GhRbohHA* | 1a | 0.59±0.16b | 0.58±0.13b | 0.57±0.13b | 0.55±0.03b |
| *GhRbohHD* | 1a | 0.43±0.15b | 0.07±0.03c | 0.28±0.07b | 0.03±0.02c |
| *GhRbohEA* | 1c | 1.17±0.06c | 0.71±0.04d | 5.10±1.16b | 13.83±4.90a |
| *GhRbohED* | 1b | 0.95±0.17b | 1.52±0.23a | 1.23±0.21ab | 1.57±0.10a |
| *GhRbohF-1A* | 1d | 2.85±0.77c | 5.52±1.26b | 9.70±1.53a | 10.70±1.71a |
| *GhRbohF-2A* | 1c | 1.16±0.15c | 1.54±0.17b | 1.11±0.12c | 5.78±0.42a |
| *GhRbohF-3A* | 1d | 1.71±0.23c | 2.25±0.01b | 2.36±0.06b | 7.83±0.81a |
| *GhRbohK-1D* | 1c | 1.17±0.13b | 1.50±0.06a | 1.42±0.10a | 1.61±0.15a |
| *GhRbohK-2A* | 1b | 1.94±0.24a | 2.22±0.31a | 2.97±0.58a | 2.31±0.29a |
| *GhRbohK-3A* | 1d | 1.59±0.15c | 3.20±0.45b | 3.53±0.24b | 6.00±0.73a |

Table S5 Effects of 10% PEG on the expression of *GhRbohs* in roots

Note: *GhUBQ7* was applied as the internal control. The expression value at 0 h was set as 1. Data are presented as mean ± SE. Different lowercase letters above the error bar indicate significant differences in gene transcription levels at different time points by one-way ANOVA and Tukey’s HSD test (n≥3, *P* ≤ 0.05).

| Genes | 0 h | 3 h | 6 h | 12 h | 24 h |
| --- | --- | --- | --- | --- | --- |
| *GhRbohEA* | 1b | 0.18±0.02c | 1.02±0.11b | 2.11±0.37a | 2.42±0.29a |
| *GhRbohF-1A* | 1a | 0.05±0.01c | 0.07±0.01c | 0.33±0.07b | 0.24±0.04b |
| *GhRbohF-1D* | 1b | 0.31±0.06c | 0.30±0.10c | 0.24±0.12c | 1.37±0.12a |
| *GhRbohF-2D* | 1a | 0.13±0.01c | 0.08±0.01d | 0.19±0.09b | 0.37±0.03b |
| *GhRbohF-3A* | 1a | 0.29±3.47E-4c | 0.65±0.09b | 0.09±0.06d | 1.01±0.11a |
| *GhRbohF-3D* | 1a | 0.54±0.03b | 0.56±0.02b | 0.89±0.13a | 0.57±0.01b |
| *GhRbohHD* | 1d | 0.32±0.17e | 2.34±0.43c | 3.74±0.37b | 7.01±0.69a |
| *GhRbohK-2D* | 1a | 0.21±0.05c | 0.37±0.10b | 0.49±0.09b | 0.72±0.21a |
| *GhRbohLA* | 1a | 0.45±0.05c | 0.21±0.02d | 0.89±0.06ab | 0.69±0.18b |
| *GhRbohLD* | 1b | 0.36±0.01c | 0.30±0.01c | 1.18±0.11b | 2.14±0.23a |
| *GhRbohBA* | 1d | 7.56±0.70a | 6.77±0.80a | 4.08±0.04c | 5.31±0.47b |
| *GhRbohBD* | 1c | 6.85±0.32a | 5.97±0.34a | 4.12±0.39b | 4.84±0.16b |
| *GhRbohD-1A* | 1a | 1.02±0.02a | 1.27±0.29a | 0.16±0.05c | 0.33±0.02b |
| *GhRbohD-2D* | 1d | 9.29±1.48a | 1.53±0.37c | 1.16±0.22d | 2.60±0.25b |
| *GhRbohF-2A* | 1b | 3.00±1.11a | 0.88±0.12b | 0.53±0.06c | 0.23±0.08d |
| *GhRbohHA* | 1a | 1.05±0.35a | 1.04±0.33a | 0.27±0.01b | 0.26±0.10b |
| *GhRbohK-1A* | 1d | 3.83±0.17a | 2.47±0.05b | 1.65±0.13c | 1.44±0.26c |
| *GhRbohD-1D* | 1a | 0.68±0.12b | 0.52±0.01b | 0.18±0.01c | 0.23±0.01c |
| *GhRbohD-2A* | 1a | 0.30±0.15d | 0.84±0.01b | 0.53±0.10c | 0.60±0.10c |
| *GhRbohED* | 1a | 0.92±0.02b | 0.69±0.16c | 0.24±0.02d | 0.86±0.19a |
| *GhRbohK-2A* | 1a | 0.05±0.01c | 0.12±0.01c | 0.10±0.01c | 0.30±0.01b |
| *GhRbohD-3A* | 1b | 1.71±0.29a | 1.47±0.18a | 1.74±0.43a | 1.94±0.46a |
| *GhRbohD-3D* | 1c | 1.12±0.10c | 3.11±0.27b | 0.40±0.08d | 6.75±2.47a |
| *GhRbohK-1D* | 1e | 1.27±0.02d | 1.59±0.38c | 2.42±0.26b | 3.67±0.82a |
| *GhRbohK-3A* | 1c | 0.77±0.13d | 2.69±0.39b | 4.71±1.25a | 5.54±1.59a |
| *GhRbohK-3D* | 1b | 0.77±0.21b | 3.94±0.39a | 3.60±1.11a | 4.40±1.13a |

Table S6 The relative expression of *GhRbohs* under heat stress in leaves

Note: *GhUBQ7* acts as the internal control. The relative expression value at 0 h was set as 1. Data are presented as mean ± SE. Different lowercase letters above the error bar show significant differences in gene expression levels at different time points by one-way ANOVA and Tukey’s HSD test (n≥3, *P* ≤ 0.05).

| Genes | 0 h | 3 h | 6 h | 12 h | 24 h |
| --- | --- | --- | --- | --- | --- |
| *GhRbohBD* | 1a | 0.84±0.09a | 0.34±0.11b | 0.28±0.09b | 0.92±0.07a |
| *GhRbohD-1A* | 1b | 0.45±0.08c | 0.42±0.02c | 0.18±0.02d | 1.69±0.13a |
| *GhRbohD-3A* | 1a | 0.09±0.02c | 0.10±0.05c | 0.04±0.01c | 0.32±0.14b |
| *GhRbohD-3D* | 1b | 0.36±0.11c | 0.49±0.12c | 0.28±0.09c | 2.25±0.13a |
| *GhRbohF-3A* | 1a | 0.54±0.02c | 0.41±0.11c | 0.17±0.07d | 0.81±0.04b |
| *GhRbohBA* | 1c | 1.14±0.30c | 1.53±0.31b | 0.17±0.05d | 4.58±0.29a |
| *GhRbohD-1D* | 1b | 2.38±0.50a | 2.06±0.23a | 0.74±0.20b | 2.93±0.40a |
| *GhRbohD-2A* | 1b | 0.98±0.20b | 1.70±0.19a | 1.45±0.10a | 0.84±0.06b |
| *GhRbohD-2D* | 1b | 1.40±0.10a | 1.07±0.01b | 1.13±0.05b | 1.30±0.11a |
| *GhRbohEA* | 1b | 1.46±0.02a | 0.49±0.03c | 0.31±0.05d | 0.37±0.01d |
| *GhRbohED* | 1c | 2.03±0.39a | 0.86±0.11c | 0.30±0.10d | 1.53±0.17b |
| *GhRbohF-1A* | 1d | 6.05±0.87b | 1.98±0.18c | 1.17±0.39d | 10.67±1.41a |
| *GhRbohF-1D* | 1c | 8.13±1.01a | 5.64±0.27b | 1.10±0.19c | 0.77±0.10d |
| *GhRbohF-2D* | 1b | 0.63±0.12c | 3.87±0.38a | 0.08±0.01e | 0.40±0.01d |
| *GhRbohF-3D* | 1c | 1.45±0.29b | 1.12±0.20c | 0.23±0.06d | 3.33±0.72a |
| *GhRbohHA* | 1b | 7.21±1.56a | 1.12±0.20b | 0.90±0.01b | 7.55±0.94a |
| *GhRbohHD* | 1e | 5.94±1.30b | 3.26±0.79c | 1.91±0.37d | 17.27±4.41a |
| *GhRbohK-2A* | 1b | 1.13±0.14b | 3.13±0.39a | 0.33±0.08c | 0.37±0.01c |
| *GhRbohK-2D* | 1c | 1.66±0.14b | 3.29±0.19a | 0.41±0.08d | 0.89±0.22c |
| *GhRbohK-3A* | 1c | 4.10±1.24ab | 3.23±0.02b | 0.55±0.10d | 5.62±0.37a |
| *GhRbohK-3D* | 1b | 1.32±0.06a | 0.40±0.06d | 0.15±0.04e | 0.73±0.07c |
| *GhRbohLA* | 1c | 2.54±0.15b | 3.43±1.05ab | 0.73±0.10d | 4.76±1.21a |
| *GhRbohLD* | 1b | 1.18±0.09b | 3.15±0.58a | 0.51±0.24c | 3.17±0.47a |
| *GhRbohK-1A* | 1a | 0.47±0.10b | 0.33±0.11bc | 0.13±0.11c | 0.15±0.11c |
| *GhRbohK-1D* | 1a | 0.25±0.06b | 0.19±0.01b | 0.08±0.01c | 0.13±0.01c |
| *GhRbohF-2A* | 1c | 1.16±0.13c | 1.54±0.15b | 1.11±0.10c | 7.77±1.98a |
